# Attitude of Basic Science Medical Students Toward Interprofessional Collaboration

**DOI:** 10.7759/cureus.333

**Published:** 2015-09-25

**Authors:** P Ravi Shankar, Neelam R Dwivedi, Atanu Nandy, Ramanan Balasubramanium

**Affiliations:** 1 Pharmacology, Medical Education, Xavier University School of Medicine; 2 Clinical Medicine, Xavier University School of Medicine; 3 Microbiology, Xavier University School of Medicine; 4 Pathology, Xavier University School of Medicine

**Keywords:** attitude, caribbean, interprofessional collaboration, interprofessional education, medical students

## Abstract

Purpose: Interprofessional collaboration (IPC) and interprofessional education (IPE) are increasingly emphasized in the education of health professions. Xavier University School of Medicine, a Caribbean medical school admits students from the United States, Canada, and other countries to the undergraduate medical course. The present study was carried out to obtain information about the attitude toward IPC among basic science medical students and note differences, if any, among different subgroups.

Methods: The study was conducted among first to fifth semester students during July 2015 using the previously validated Jefferson Scale of Attitudes Toward Interprofessional Collaboration (JeffSATIC). Gender, age, semester, and nationality were noted. Participants’ agreement with a set of 20 statements was studied. Mean total scores, working relationship, and accountability scores were calculated and compared among different subgroups of respondents (p<0.05).

Results: Sixty-seven of the 71 students (94.4%) participated. Cronbach’s alpha value of the questionnaire was 0.827, indicating good internal consistency. The mean total score was 104.48 (maximum score 140) while the working relationship and accountability scores were 63.51 (maximum score 84) and 40.97 (maximum score 56), respectively. Total scores were significantly higher among third-semester students and students of Canadian nationality. Working relationship and accountability scores were higher among first and third-semester students.

Conclusion: The total working relationship and accountability scores were lower compared to those obtained in a previous study. Opportunities for IPE and IPC during the basic science years should be strengthened. Longitudinal studies in the institution may be helpful. Similar studies in other Caribbean medical schools are required.

## Introduction

Interprofessional collaboration (IPC) has been defined as ‘the process of developing and maintaining effective interpersonal working relationships with learners, practitioners, patients, clients, families, and communities to enable optimal health outcomes’ [1]. Interprofessional learning (IPL) arises from interactions between the members (or students) of two or more professions and may result from interprofessional education or may happen in a spontaneous manner in the workplace, education, or other settings [2]. The World Health Organization (WHO) defines IPC as ‘multiple health workers from different professional backgrounds, provide comprehensive services by working with patients, their families, caregivers, and the community to deliver the highest quality of care across settings’ [3].

Teamwork and collaboration among healthcare providers are increasingly regarded as an important component of professionalism. The authors of a recent article describe the key concepts in most descriptions of IPC as ‘shared responsibilities’, ‘communication’, ‘accountability’, ‘shared decisions’, and ‘education’ [4]. Collaboration is important in today’s healthcare environment and its practice has been deemed essential for positive patient outcomes [5]. IPE has been recommended to improve IPC based on the hypothesis that if individuals from different professions learn together, they and their agencies will work better together, resulting in improved care and delivery of service [5]. A systematic review identified 10 challenges and barriers in implementing IPE in developed countries ranging from the curriculum of different healthcare students, leadership, resources, stereotypes and attitudes of faculty members, variety of students, differences in the concept and methodology of IPE programs, challenges in teaching IPE, lack of enthusiasm, professional jargon, and accreditation issues [6].   

Xavier University School of Medicine is a Caribbean medical school situated in Aruba, Kingdom of the Netherlands admitting students from the United States (US), Canada, and other countries to the undergraduate medical (MD) course. Students complete the first six semesters of study in Aruba and then do their clinical rotations in the US or Canada. Like in most other Caribbean schools, a semester is of 15 weeks duration. Recently, the school shifted to an integrated, organ system-based curriculum during the basic science years and early clinical exposure has been introduced [7]. During their hospital observership and in the clinics of the general practitioner, students interact with nurses and other healthcare professionals; however, opportunities for IPE and IPC are currently limited during the basic science years. The school is in the process of initiating a Bachelor of Nursing program which may provide greater opportunities for IPE and IPC. Student attitudes towards IPC have not been previously studied in the institution. Hence, the present study was conducted to study the attitude towards IPC among undergraduate basic science medical students in the institution and note differences, if any, in the attitude among different subgroups of respondents.

## Materials and methods

The study was approved by the Institutional Review Board of Xavier University School of Medicine vide notification XUSOM/IRB/2015/03.

The present study was conducted among the first to fifth-semester basic science undergraduate medical (MD) students at the institution during July 2015. Their attitudes towards interprofessional collaboration were studied using the previously validated Jefferson Scale of Attitudes Toward Interprofessional Collaboration (JeffSATIC) developed by Hojat and coworkers [4] after obtaining written permission from the developers. The instrument was developed considering the fact that previous instruments were developed for use among specific populations and some of the instruments had been used only once by their developers, were not developed on the basis of principles of test construction, and were not supported by convincing psychometric evidence. JeffSATIC measures attitudes towards interprofessional collaboration by noting respondents’ agreement with a set of 20 statements using a Likert-type scale from 1 to 7. Definitions of important terms in the questionnaire are provided and demographic details can be added as needed. The instrument was developed according to sound psychometric principles and has been pretested and validated in a recent study [4]. 

Students were informed of the objectives of the study and invited to participate. Written informed consent was obtained from all participants. Gender, age in years, the semester of study, and nationality was noted. On reviewing the list of our students, we observed we had some young students (less than 20 years) who had joined the MD program after completing the approximately two-year pre-medical course either at Xavier University or other universities. We had students who joined after completing a graduate course of study and who were aged between 20 to 25 years. We also had more senior students who had joined after spending a few years working in other professions and were aged over 25 years. The division of ages hence corresponds to the distribution of the student ages. Participants’ agreement with a set of 20 statements was noted using a 7 point scale ranging from 1 – strongly disagree with the statement to 7 – strongly agree with the statement. The scores of statements 3, 5, 8, 9, 12, 15, 16, and 19 were reversed while calculating the total score and subscale scores. The maximum possible score was 140. The higher the score the greater was the orientation toward teamwork and collaboration. One sample Kolmogorov-Smirnov (KS) test was used to assess the normality of the distribution of the scores of individual statements and the total score. Cronbach’s alpha was calculated as a measure of internal consistency of the questionnaire. The average total score was calculated and compared among different subgroups of respondents using appropriate statistical tests. A p value of less than 0.05 was taken as statistically significant. As recommended by the developers of the instrument, two constructs, ‘working relationship’ and ‘accountability’, were also calculated. A working relationship was the sum of scores of statements 1 to 12 while accountability was the sum of the scores of statements 13 to 20. The normality of distribution of the scores of these two constructs was examined using one sample K-S test. The average scores were compared among different subgroups of respondents. Free text comments from the respondents were invited and were tabulated.

## Results

Sixty-seven of the 71 students (94.4%) participated in the study. Table 1 shows the demographic characteristics of the respondents. A greater number of respondents were female, in the age group between 21 to 25 years, and of American nationality.

**Table 1:** Demographic characteristics of the respondents

Characteristic	Number (percentage)
Gender	
Male	30 (44.8)
Female	36 (53.7)
Age	
Less than or equal to 20	15 (22.4)
21 to 25	33 (49.3)
Greater than 25	16 (23.9)
Semester of study	
First	19 (28.4)
Second	14 (20.9)
Third	17 (25.4)
Fifth	17 (25.4)
Nationality	
American	28 (41.8)
Canadian	18 (26.9)
Others	18 (26.9)

Cronbach’s alpha value for the questionnaire was 0.827, which indicates a good level of internal consistency. On carrying out the one sample Kolmogorov-Smirnov test, it was noted that the majority of the individual scores were not normally distributed while the total score was normally distributed. Table 2 shows the median score of individual statements and the mean total score.

**Table 2:** Average scores of individual statements and the total score

Statement	Average Score
One	5
Two	6
Three	4
Four	5
Five	6
Six	6
Seven	7
Eight	5
Nine	4
Ten	7
Eleven	6
Twelve	5
Thirteen	6
Fourteen	6
Fifteen	4
Sixteen	4
Seventeen	6
Eighteen	6
Nineteen	4
Twenty	6
Total	104.48

The total score was obtained as described in the Materials and Methods section. The reversed score of statements 3, 9, 15, 16, and 19 were low. The mean total score was 104.48 (maximum possible score being 140).

Table 3 shows the mean total scores among various subgroups of respondents. The score was higher among female respondents and respondents over the age of 25 years, but the difference was not significant. The scores were significantly lower among the second-semester students and were the highest among the third-semester students. The score was highest among students of Canadian nationality.

**Table 3:** Mean total scores among different subgroups of respondents

Characteristic	Mean Score	P value
Gender		
Male	102.8	0.524
Female	105.17	
Age		
Less than or equal to 20	98.07	0.075
21 to 25	105.18	
Greater than 25	110.06	
Semester of study		
First	105.58	0.004
Second	94.5	
Third	113.70	
Fifth	102.23	
Nationality		
American	103.36	0.079
Canadian	109.33	
Others	98.06	

The mean total ‘Working relationship’ score was 63.51 (maximum possible score being 84) while the ‘Accountability’ score was 40.97 (maximum score being 56). Table 4 shows the mean working relationship and accountability scores among different subgroups of respondents. Both scores were significantly higher among the first and third semester students and the working relationship score was higher among Canadian students.    

**Table 4:** Mean working relationship and accountability scores among different subgroups of respondents

Characteristic	Mean Working Relationship Score	P value	Mean Accountablity Score	P Value
Gender				
Male	62.43	0.529	40.37	0.611
Female	63.97		41.19	
Age				
Less than or equal to 20	59.87	0.112	38.2	0.106
21 to 25	63.88		41.30	
Greater than 25	67.12		42.94	
Semester of study				
First	63.90	0.004	41.68	0.033
Second	57.00		37.50	
Third	69.59		44.12	
Fifth	62.35		39.88	
Nationality				
American	63.18	0.045	40.18	0.529
Canadian	67.17		42.17	
Others	59.05		40.00	

Among the free text comments were ‘Coordination and collaboration between different disciplines in healthcare are beneficial and essential to optimal patient care’, ‘This is a very interesting topic that I find many people do not notice as much. We need to learn to work in teams in our own fields/professions but also with other professions that have the same goal as us – delivering the best quality care to patients’.

## Discussion

The mean total score in the present study was 104.48 while the working relationship and accountability scores were 63.51 and 40.97. Differences in these scores were noted among certain subgroups of respondents. JeffSATIC is a recently developed scale and previous studies using the instrument is limited. In a multi-institutional study conducted to validate the instrument, the scores were higher compared to the present study [4]. The study was conducted at Jefferson and Midwestern University in the US and Monash University in Australia. The mean total scores were 119.3 at Jefferson, 119.4 at Midwestern, and 114.2 at Monash University. The working relationship scores were 74.3 at Jefferson, 75.7 at Midwestern, and 71.6 at Monash. With regard to accountability scores, the values at Jefferson, Midwestern, and Monash were 45.0, 43.7, and 42.7, respectively. In all three institutions, the scores were higher among female respondents [4]. There was no significant difference in scores among different age groups of respondents. At XUSOM, opportunities for IPC and IPE are limited; many students, though from the US and Canada, were of Asian origin, which may be responsible for the lower score. The low score of the second-semester students may have influenced the total score.        

A previous study using the Jefferson Scale of Attitudes toward Physician-Nurse Collaboration found that female students had a more positive attitude towards collaboration [8]. There were no differences noted among first and final year medical students, and students who had an interprofessional thread within their medical curriculum did not show a more favorable attitude towards collaboration. In the present study, differences were noted among the different semesters of students with the second-semester students having a lower score. Differences were also noted according to nationality in certain scores. Canadian students had the highest scores while students from other countries had lower scores. The ‘other countries’ group is diverse with students from Latin America, Africa, and Asian countries. Students from developed nations may have a more positive attitude toward interprofessional collaboration, though many of these students were of Asian origin.

A study conducted among physicians and nurses in the US and Mexico using the Jefferson Scale of Attitudes Toward Physician-Nurse Collaboration found that physicians and nurses in the US had more positive attitudes towards collaboration [9]. Also, nurses in both countries had more positive attitudes towards collaboration compared to physicians, but female physicians did not show a more positive attitude toward collaboration compared to their male counterparts. Students from six disciplines at Monash University in Australia completed the Readiness for Interprofessional Learning Scale before participating in interprofessional clinical learning modules [10]. Nursing students had more positive attitudes towards teamwork and collaboration, and one-third of all students who had prior experience of interprofessional learning held more positive attitudes. We only studied attitudes towards IPC among medical students as other categories of students are not enrolled in the institution.

Medical and nursing students at two universities in Sweden completed the Readiness for Interprofessional Learning Scale [11]. The results showed female students had a more positive attitude towards teamwork and collaboration compared to male students. Nursing students had a more positive attitude towards collaboration. The increasing number of female medical students in recent years could have implications towards collaboration and teamwork. A study conducted in both Iran and Germany explored health care practitioners' (including medical doctors, nurses, social workers, and psychologists) experiences and perceptions of the challenges of IPC [12]. The results showed that in both the eastern and western context, organizational, professional, and community sociocultural factors in terms of attitude towards other people, other professions, and IPC played an important role. Readiness for IPE and IPC has been traditionally studied in developed nations. IPE is strongest in the United Kingdom, Canada, Australia, and Japan [11]. Information from the developing world is less. However, a study among students at the National University of Singapore showed that overall, students had a high readiness for IPE [13]. However, the readiness was lower among pharmacy and dentistry students compared to medical students. The readiness for interprofessional learning scale has been validated among health professions students in Indonesia [14] and the United Arab Emirates [15]. Researchers may use the validated instrument to study readiness for IPL in developing nations in future.

Many accrediting and regulatory bodies require health profession schools to offer IPE and promote IPC among students. The academic leadership at XUSOM is in favor of offering opportunities for IPE and IPC to students. Starting off the nursing program will offer opportunities for both. Like in other Caribbean schools, students do their clinical rotations at different hospitals in the US and Canada, and many of these institutions do emphasize IPE. We did not study attitude toward IPC among the clinical students at the institution. Interprofessional education programs could be considered during the basic science and clinical years of study at the institution. A controlled trial of an eleven-hour IPE program was carried out at a university in New Zealand [16]. The intervention improved student attitudes toward interprofessional teams and interprofessional learning. Students’ self-reported ability to function within an interprofessional team also improved. As a majority of XUSOM graduates will do their clinical rotations and practice in the US and Canada, student attitude towards IPE and IPC will have an influence in these locations. Caribbean medical schools provide a significant proportion of international medical graduates (IMGs) in the United States [17], and around 20% of Canadian IMGs had obtained their medical degree from the Caribbean [18]. Thus, graduates from Caribbean medical schools make an important contribution to the health workforce in these countries, and studies on attitudes toward IPC in other Caribbean medical schools are required.

The limitation of the present study was that student attitude toward interprofessional collaboration was studied only using a previously validated questionnaire. The data obtained was not triangulated with that obtained using other instruments or sources.

## Conclusions

The total working relationship and accountability scores were lower compared to those obtained in a previous study. Differences in scores among different subgroups of respondents were noted. At present, opportunities for IPE and IPC during the basic science years are less in the institution and should be strengthened. Longitudinal studies in the institution may be helpful. Similar studies in other Caribbean medical schools are required.
